# Mild hypothermia (35°C) reduces myocardial ischemia-reperfusion injury and attenuates hypoxia induced apoptosis of H9C2 cardiomyocytes by changing the phosphorylation level of Connexin43 (Cx43) protein

**DOI:** 10.4314/ahs.v25i1.18

**Published:** 2025-03

**Authors:** Yuanming Lu, Fang Wang, Nanping Xiao, Zilian Fan, Lan Xiong, Jing Kou, Tianxun Wang, Dianyuan Li

**Affiliations:** 1 Department of Neurology, First People's Hospital of Guangyuan, Guangyuan, China; 2 Drug Supply Station, Chinese people's liberation army 32603 troops, Chengdu, China; 3 Department of Gastroenterology, First People's Hospital of Guangyuan, Guangyuan, China; 4 Department of Cardiology, First People's Hospital of Guangyuan, Guangyuan, China; 5 Department of Cardiothoracic Surgery, The Affiliated Suzhou Hospital of Nanjing Medical University, Suzhou Municipal Hospital, Gusu School, Nanjing Medical University, Suzhou, China

**Keywords:** Mild hypothermia, Cx43, ischemia reperfusion, hypoxia, apoptosis

## Abstract

**Background:**

The purpose of this study was to investigate the effect of connexin 43 (Cx43) on myocardial cell apoptosis under mild hypothermia and its potential for treating ischemia reperfusion injury.

**Methodology:**

In vivo experiments were conducted on rats, using an ischemia-reperfusion model, small animal ultrasound imaging system, and relevant biochemical assays to measure myocardial function, infarction area, and tissue damage. In vitro experiments were performed on H9C2 cells using an oxygen-glucose deprivation and recovery model, and various assays were used to assess cell viability, apoptosis, and biochemical changes.

**Results:**

Mild hypothermia (35°C) was found to reduce ischemia-reperfusion injury, enhance myocardial function, decrease infarction area, and increase the expression of phosphorylated Cx43 and protein kinase C in myocardial tissue. In vitro, mild hypothermia enhanced cell viability, decreased gap junction permeability, downregulated pro-apoptotic factors, and upregulated anti-apoptotic factors, while also increasing the levels of calcium and superoxide dismutase and decreasing the level of malondialdehyde.

**Conclusions:**

Mild hypothermia can protect against myocardial ischemia-reperfusion injury by regulating the level of phosphorylated Cx43 protein, which reduces myocardial cell apoptosis and enhances cardiac function. This study highlights the potential therapeutic benefits of mild hypothermia in treating ischemia-reperfusion injury.

## Introduction

Myocardial ischemia-reperfusion injury (MIRI) is a condition in which a patient returns to blood supply after a coronary artery obstruction, but the Myocardial ischemia injury is more severe than before^1^. Ischemia reperfusion have multiple effects on myocardial injury, including myocardial edema, myocardial hemorrhage, ultrastructural changes, intracellular accumulation of myocardial enzymes, leakage of myocardial enzymes, and myocardial oxygenation disorders^2^. Although drug therapy has been applied according to different mechanisms of ischemia reperfusion injury in clinical practice, such as melatonin, adenosine, metoprolol, and abciximab, the efficacy is relatively limited. Therefore, the prevention and treatment of myocardial ischemia reperfusion injury is still a difficult problem in the field of cardio-vascular medicine. It is imperative to explore the pathogenesis of myocardial ischemia reperfusion injury, establish new effective treatment measures, and reduce the incidence of myocardial ischemia reperfusion injury in patients.

Connexin 43 is a transmembrane protein that exists in a specific region of the cell membrane in the form of hexamer^3^. At present, CX43 has been confirmed to be distributed in 34 kinds of tissues and 46 types of cells. Under normal conditions, a gap junction (GJ) is formed between two adjacent cells to realize substance exchange and signal transmission between cells. This special membrane structure plays an important role in mediating cardiac electrical coupling, realizing synchronous contraction of atrium or ventricle and ensuring normal rhythm of heart^4^,^5^. The other part exists directly in the cell membrane to form a semi-channel that allows material exchange and signal transduction inside and outside the cell in the form of ligons, and regulates cell volume and activates cell survival pathways under physiological or ischemic conditions^6^.

Phosphorylation of Connexin 43 plays an important role in gap junction formation and stability, channel function, and disease development, and is phosphorylated in different ways throughout the life cycle. TPA 12-o-tertradecanoylphordol-13-acetate, TPA and Protein kinase C can phosphorylate S368 of CX43, resulting in gap junction closure and reduced intercellular connectivity^7^. In addition, MAPK pathway activates phosphorylation of S255, S262, S282and S279 in CX43 protein, which can also reduce gap junction opening^8^. In the normal physiological state, CX43 is in the phosphorylated state, at which point the GJ channel is closed, but dephosphorylation during ischemia/reperfusion injury causes the GJ channel to open^9^,^10^. smission between adjacent cells^11^. Therefore, CX43 can be used as a molecular target for the protection or treatment of myocardial ischemia reperfusion injury, and has objective research value.

A number of studies have shown that even a small temperature decrease can have a protective effect on the beating heart and effectively prevent the occurrence of myocardial infarction, and it is positively correlated with the degree of temperature decrease^12^,^13^. Animal experiments have shown that each 1°C reduction in core temperature can reduce the size of myocardial infarction about 67%, so that the myocardial ischemia reperfusion injury during acute myocardial infarction can be protected, and this study also suggests that hypothermia therapy is an effective cardiac protection strategy for myocardial infarction. In the past, studies related to hypothermia in the treatment of myocardial ischemia reperfusion injury focused on hypothermia below 30°C or more deep hypothermia. However, it has been found that the tolerance of animal cells to low temperature is limited, and too low temperature can cause problems such as hypotension, arrhythmia and blood viscosity^14^. Therefore, the reduction of temperature must be controlled within a certain range to ensure the effectiveness of treatment, so as to avoid the occurrence of other symptoms. Hypothermia represents temperatures in the range of 32°C to 35°C. Although this temperature range can affect myocardial cell contraction and cardiac function, there is no obvious adverse reaction, which proves that it can be used as an effective temperature for hypothermia treatment of myocardial diseases^12^,^14^,^15^. Mild hypothermia affects the expression of Hippocampal connexin 43 and Glutamate transporter 1 after traumatic brain injury^16^. Short-time hypothermia therapy can induce and promote connexin 43 gap junction reconstruction in isolated rabbit hearts^17^. Hypothermia therapy (TH) increases susceptibility to ventricular arrhythmias by reducing ventricular conduction velocity (CV) and promoting arrhythmogenic spatial incongruous alternation (SDA)^18^. These results all prove that mild hypothermia is closely related to the functional changes of connexin proteins.

Based on the above mechanism of connexin in myocardial cells and the research progress of mild hypothermia in protecting animal myocardium from ischemia reperfusion injury, we speculate that mild hypothermia may play a protective role in myocardium from ischemia reperfusion injury by acting on connexin Cx43. This study will reveal the regulatory mechanism of 35°C mild hypothermia and Cx43 protecting myocardial ischemia-reperfusion injury through experiments, which may also provide a new therapeutic strategy.

## Materials and Methods

### Surgical procedure of myocardial I/R model

The left anterior descending (LAD) coronary artery was ligated, the ligature line was loosened, and the myocardial ischemia reperfusion model was established^19^. Rats were anesthetized with isoflurane (H19980141, Yipin Pharmaceutical, Guiyang, China) through a mask, and then were fixed on a board for experiment. Routine disinfection, endotracheal intubation with respirator (RWD407, Rayward, China) ventilation (frequency 70 times/min, inspiration: expiration=1:1.5), and observation of good chest lifting as a sign of successful assisted respiration. Open the chest on the left side beside the sternum, cut a skin incision of about 1.5 cm along the third and fourth ribs of the left sternal sideline, expand the third and fourth ribs to both sides, open the chest, tear the open capsule, fully expose the heart, ligate the left anterior descending branch of the coronary artery with 6-0 lines 2 mm below the left atrial appendage, quickly suture the chest with 4-0 non-invasive suture needles, and then suture the muscles and skin. After 45 min of ligation, the chest was opened again and the suture was cut for reperfusion. In the sham operation group, only threading was performed at the coronary artery without ligation. This study was approved by the Ethics Committee.

### Temperature Control and measurement

Left atrial (LA) and rectal temperature were measured with a digital thermometer. After sedation and upon arrival in the operating room, rectal temperature was measured immediately and recorded as the initial temperature. After thoracotomy, but prior to coronary occlusion, temperature was immediately recorded in the LA and recorded as the “baseline” temperature. The LA and rectal temperatures were measured every 5 minutes during ischemia and every 15 minutes during reperfusion^20^.

### Animal experiment grouping

18 healthy male SD rats, weighing 120∼150 g, were provided by the Experimental Animal Center of the Suzhou Municipal Hospital and divided into three groups, with 6 rats in each group. Pseudo operation group (6 animals): animals were routinely raised throughout the process. I/R group (6 animals): myocardial ischemia for 45 minutes, reperfusion for 24 hours, and the animals were fed routinely throughout the process. I/R+35 °C treatment group (6 animals): During 45 min of myocardial ischemia, the animals were fed in a 35°C incubator, and during reperfusion, they were fed routinely.

### Carrier construction

Using the cDNA of rat cardiac tissue Cx43 as template, the target gene was inserted into pEGFP-C1 plasmid through EcoRI and BamHI digestion sites, and the overexpression vector pEGFP-C1-Cx43OE was constructed; According to the sequence of PKC in NCBI database, the target gene was inserted into PLVshRNA EGFP (2A) Puro plasmid through BamHI and Eco-RI digestion sites, and the interference vector PLVshRNA EGFP (2A) Puro PKC was constructed.

### Establishment of myocardial cell OGD/R model

Put the sugar free DMEM medium into a three gas incubator containing 95% N2 for 2 h to fully remove the oxygen in the medium, and then use the sugar free and oxygen free DMEM medium (provided by the stem cell bank of the Chinese Academy of Sciences) to culture the myocardial cells H9C2 (normally cultured for 48 hours) in an incubator containing 95% N2 and 5% CO2 for 7 h under hypoxia. After the end of the hypoxia culture, replace the sugar free DMEM medium with the sugar containing DMEM medium Incubate in 5% CO2 incubator for 0, 2, 4, 8, 12, and 24 hours respectively. Determine the best time point of H9C2 myocardial cell hypoxia reoxygenation injury model by MTT method.

### Cell experiment grouping

H9C2 is provided by Stem Cell Bank of Chinese Academy of Sciences. The construction of the hypoxia model is realized by using a three-gas incubator. By controlling the input of oxygen, nitrogen and carbon dioxide, the oxygen content is controlled by sensors. During the experimental study, the gas ratio of 5% CO2+3% O2+92% N2 was used to reduce the oxygen supply. Control group: cells were normally cultured under normal oxygen, 37°C and 5% CO_2_ during the whole process. OGD/R group: After 7 hours of incubation at 95% N2, 37°C and 5% CO_2_, the OGD/R group was incubated at normal oxygen and 5% CO_2_ for 0-24 hours respectively. Cx43OE+OGD/R treatment group: the constructed overexpression vector was transiently transfected into H9C2 cells according to Lipo2000 liposome instructions. After 24 hours of normal culture, it was cultured under hypoxia for 7 hours. After hypoxia, it was cultured normally. OGD/R+35°C treatment group: during hypoxia culture, cells were fed in a 35°C incubator, and cultured normally after hypoxia. 35°C+shPKC+OGD/R treatment group: according to Lipo2000 liposome instructions, the constructed interference vector was transiently transfected into H9C2 cells for 24 hours, and then cultured in hypoxia for 7 hours. During hypoxia culture, cells were fed in a 35°C incubator, and then cultured normally after hypoxia.

### Ultrasonic testing

At 48 h after operation, rats were anesthetized with isoflurane through a mask. The hair on the left chest of rats was carefully removed with depilatory ointment. They were fixed on the operating platform in a supine position with adhesive tape. A small amount of couplant was applied on the left chest. The probe was placed on the left chest. Small animal ultrasonic imaging systems (Vevo-1100, VisualSonics, CAN) were used for ultrasonic testing to obtain myocardial function related indicators: left ventricular end diastolic diameter (LVIDd) Left ventricular end systolic diameter (LVIDs), left ventricular end diastolic volume (LV Vol. d), left ventricular end systolic volume (LV Vol. s), left ventricular ejection fraction (EF%) and left ventricular shortening fraction (FS%).

### Detection of myocardial infarction

Myocardial infarction was assessed with 2,3,5-triphenyltetrazolium (TTC) (Amresco, 0765) staining^21^. After ultrasonic testing, the animals were killed, the hearts were quickly removed, the remaining blood was washed with ice normal saline, and stored in - 80°C refrigerator for 5 minutes. After the quick frozen heart is cut longitudinally into a 2 mm thick slice at the ligation site, the rest of the tissue is put into a cryopreservation tube and stored at - 80°C for standby after being subjected to liquid nitrogen. Put the 2 mm thin slice in 1.5% TTC solution (pH=7.4), take a constant temperature water bath at 37°C for about 5min, and shake the dye continuously during the dyeing process to make it fuly contact with the myocardium. Take photos after dyeing. The non infarcted area is red, and the infarcted area is white. The infarct area and total area were measured with Image J software, and the percentage of infarct area to total heart area was calculated finally.

### HE staining of myocardial tissue

Cardiac tissue was stained with hematoxylin and eosin (H&E) (Servicebio, G1005, Wuhan, China). After the ultrasonic detection, the animals were killed, the hearts were quickly removed, the remaining blood was washed with ice normal saline, the myocardium of rats in each group was cut longitudinally at the ligation site, and it was quickly put into 4% paraformaldehyde for 48 hours. After the conventional paraffin embedding, the following operations were completed in sequence: dewaxing and hydration, hematoxylin staining for 3-5 minutes, differentiation, back blue, eosin staining for 5 minutes, xylene transparent, and neutral gum (Zhongshan Jinqiao, ZLI-9555) was dropped for sealing, The nucleus was blue and the cytoplasm was pink or red when observed and photographed under the microscope.

### Immunohistochemistry of myocardial tissue

Myocardial tissue was stained with immunohistochemical staining kit (Shibode, SV0004, Hangzhou, China). Myocardial tissue was prepared into about 4 µM paraffin tissue sections, routine dewaxing and hydration, antigen repair, elimination of endogenous peroxidase activity, 5% BSA, incubation at 37°C for 30 min and sealing, primary antibody Cx43 (BA1727, Boside, Wuhan, China, 1:300), p-Cx43 (P00599, Boside, Wuhan, China, 1:200), PKC (BM0401, Boside, Wuhan, China, 1:50) overnight at 4°C, secondary antibody incubation at 37°C for 30 min, diaminobiphenylamine (DAB) coloration, hematoxylin re staining, dropping neutral gum for sealing, Observe the protein expression under the microscope and take photos. Complete the analysis of the positive proportion of immunohistochemistry with Image J software.

### Determination of Ca2+content

After the in vivo and in vitro model was built, the myocardial tissue and H9C2 cells were taken respectively, and the content of Ca2+was determined with the kit (Nanjing Jiancheng, C004-2, Nanjing, China). The OD610 value is monitored by using a microplate reader (Bio Rad, Hercules, CA, USA), and the calcium content (mmol/gprot) = (the average of measured OD value/the average of standard OD value) × Standard concentration/protein concentration of the sample to be tested.

### Quantitative detection of MDA

After the in vivo and in vitro model was constructed, myocardial tissue and H9C2 cells were taken respectively, and the content of MDA was determined with the kit (Nanjing Jiancheng, A0031, Nanjing, China). The OD532 value is monitored with a microplate reader (Bio Rad, Hercules, CA, USA). The content of MDA (nmol/mgprot) = (the average of measured OD value/the average of standard OD value) × Standard concentration/protein concentration of the sample to be tested.

### Total SOD activity detection

After the in vivo and in vitro model was constructed, the myocardial tissue and H9C2 cells were taken respectively, and the total SOD activity was determined with the kit (Nanjing Jiancheng, A0013, Nanjing, China). The OD450 value is monitored by the microplate reader (Bio Rad, Hercules, CA, USA), and the SOD inhibition rate (%)=(the average of the control OD value - the average of the measured OD value)/the average of the control OD value × 100; SOD activity (U/mgprot)=SOD inhibition rate (%)/50% × Dilution multiple of reaction system/protein concentration of sample to be tested.

### Western Blotting

In order to analyze the expression of half channels and apoptosis related proteins in myocardial cells, Western Blot method was used in this experiment. After the establishment of the in vitro model, the cell precipitation of each group was collected. The RIPA lysate (Beyotime, P0013, Shanghai, China) was used to extract the total protein in the cell precipitation. BSA kit (Thermo, 23225, Waltham, MA, USA) was used to determine the concentration of protein, and then protein denaturation treatment was carried out. The protein was dissolved on sodium dodecyl sulfate polyacrylamide gel (SDS-PAGE), and then electrotransferred to polyvinylidene fluoride (PVDF) membrane. 5% skimmed milk was used for sealing. First antibody Cx43 (BA1727, PhD, China, 1:300), p-Cx43 (P00599, PhD, 1:200), PKC (BM0401, PhD, 1:50), Bcl-2 (12789-1-AP, Proteintech, Rosemont, IL, USA 1:2000), Bax (50599-1-LG, Proteintech, Rosemont, IL, USA), Bad (10435-1-AP, Proteintech, Rosemont, IL, USA, 1:1500), Cleaned Caspase-3 (19677-1-AP, Proteintech, Rosemont, IL, USA, 1:1000), Cleaned Caspase-9 (10380-1-AP, Proteintech, Rosemont, IL, USA, 1:500) and β- Actin (Santa, Santa Cruz, CA, USA, 1:800) was incubated at 4°C overnight, the second antibody Goat Anti MouselgG, (H+L) (PA1-28555, Thermo, Waltham, MA, USA, 1:10000) and Goat Anti RabbitlgG, (H+L) (31210, Thermo, USA, 1:10000) were incubated at room temperature for 90 min, developed with chemiluminescent substrate (32209, Thermo, Waltham, MA, USA), scanned the film, and integrated the result map.

### QPCR detection

After the establishment of the in vitro model, collect the cell precipitation of each group, add 1 ml TRIzol (9109, TaKaRa, Tokyo, Japan), repeatedly blow and suck until there is no obvious precipitation in the lysate, and let it stand at room temperature for 5 minutes. Add 200 µl of chloroform, shake violently for 15 seconds, stand at room temperature for 3-5 min, and centrifugate 12000 g at 4°C for 15 min. Suck up the upper clear water phase and add isopropyl alcohol of equal volume, invert it and fully mix it, then let it stand at room temperature for 10 min, and centrifugate it at 4°C for 10 min at 12000 g. Discard the supernatant and wash it twice with 75% ethanol precooled at - 20°C. Discard ethanol, and dry at room temperature in the ultra clean workbench for 5 min. Add 20∼50 µl RNase free water to dissolve the precipitate, and incubate it in a 55°C water bath for 10 min. After RNA precipitation is completely dissolved, use 1% agarose gel electrophoresis to verify its integrity, use NanoDrop 2000c nucleic acid quantitative detector (Thermo, Waltham, MA, USA) to detect its concentration and purity, and store qualified samples at - 80°C. Using PrimeScript ™ RT reagent Kit with gDNA Eraser (Perfect Real Time) (RR047A, TaKaRa, Tokyo, Japan) kit is used to reverse transcript samples with complete structure, and the reverse transcripted cDNA is stored at - 20°C. Use SYBR ® Premix Ex Taq ™ II (Tli RNaseH Plus) (CW0957A, Kangweishiji, Taizhou, China) kit uses Mastercycler ep realtime quantitative PCR instrument (Eppendorf, Germany) to perform qPCR detection according to the following procedures: first 95°C for 10 min, then 35 cycles: 95°C for 10 s, 58°C for 30 s, and finally 72°C for 32 s. Specific primers used for qRT PCR are shown in Table 1. Use 2–ΔΔCt method normalized the target gene level to GAPDH level. As shown in Table 1.

**Table 1:** The primers of detected mRNA

Primers (5′→3′)		
Cx43	Forward	GGAAAGTACCAAACAGCAGCA
	Reverse	CAAAGTTGGTGGAACTCCTTG
PKC	Forward	TGCCTGCTCCAGACTAAGAT
	Reverse	CCCAGCCTCCCACTTAATTC
Bcl-2	Forward	ACCCCTTCATCCAAGAATGC
	Reverse	TACCAATAGCACTTCGCGTC
Bad	Forward	TTGAGGAAGTCCGATCCCG
	Reverse	GAACATACTCTGGGCTGCTG
Bax	Forward	ACGTCTGCGGGGAGT
	Reverse	GAAACCCTGTAGCAAAAAGGC
Cleaved Caspase-3	Forward	GGGAGCTTGGAACGCTAAG
	Reverse	TGCATATGCCCATTTCAGGA
Cleaved Caspase-9	Forward	GAAAAGTGGCTCCTGGTACAT
	Reverse	CAGCCAGGAATCTGCTTGTAA
GAPDH	Forward	TGTGTCCGTCGTGGATCTGA
	Reverse	TTGCTGTTGAAGTCGCAGGAG

### MTT detection

The viability of H9C2 cells was detected with MTT kit (C0009S, Beyotime, Shanghai, China). After deprivation of oxygen and sugar for 7 hours, respectively restore oxygen and sugar for 0 h, 2 h, 4 h, 8 h, 12 h, 24 h, add 20% to each hole µ L MTT solution (5 mg/mL, dissolved in fresh serum free medium), and incubated at 37°C for 4 h. After removing the supernatant, add 150 µL Dimethyl sulfoxide (DMSO) to completely dissolve the formed formazan crystal. The OD490 value was monitored by using a microplate reader (Bio Rad, Hercules, CA, USA). Cell survival rate%=(average OD value of experimental group/average OD value of control group) × 100%.

### TUNEL detection

TUNEL was used to detect apoptosis of H9C2 cells. After oxygen sugar deprivation for 7 hours and oxygen sugar recovery for 24 hours, the culture medium was aspirated and discarded, 0.01 mol/L PBS (pH7.4) was washed for 2-3 times, and then 4% paraformaldehyde was used to fix each well at room temperature for 30 minutes, PBS was washed for 2-3 times, 0.3% Triton X-100 was used to incubate at room temperature for 15 minutes, and PBS was washed for 2-3 times. Then TUNEL test kit (G3250, Promega, Madison, WI, USA) was used to detect DNA fragments broken during apoptosis. Dye the nucleus with DAPI (AR1177, Boshi De, Wuhan, China), observe the apoptotic cells under fluorescence microscope and take photos after the film is sealed, and count the number of apoptotic cells with Image-J software.

### LY detection

The gap junction permeability of H9C2 cells was measured by diffusion distance of LY staining. After oxygen glucose deprivation for 7 hours and recovery for 24 hours, the cells were washed with 0.01 mol/L PBS (pH7.4) at 37°C for three times. Add a small amount of pretreated 0.05% LY dye (L4042, uebio, China) 2 mL. Scratch the cell surface of the plastic culture dish with a sharp surgical blade, mark it for 5 min, and suck out the LY dye. PBS was washed 3 times to remove free fluorescent dyes and exfoliated cells. Add 4% paraformaldehyde solution to fix the cells for 30 min. Observe the fluorescence transmission under the fluorescence microscope and take photos. Use Image-J software to measure the distance of fluorescence transmission on both sides of the notch.

### Statistics analysis

All the tests in this study were 3 replicates, and all the data were expressed as mean ± standard deviation (SD). GraphPad Prism 8 software (La Jolla, CA, USA) was used for data analysis. Single factor (single factor and multiple levels) or two factor (two factor and multiple levels) analysis of variance (ANOVA) was used for comparison between groups, and Tukey test was used. Use */# to indicate that the difference is statistically significant (*/# p<0.05, * */# p<0.01).

## Result

**Figure 1:**
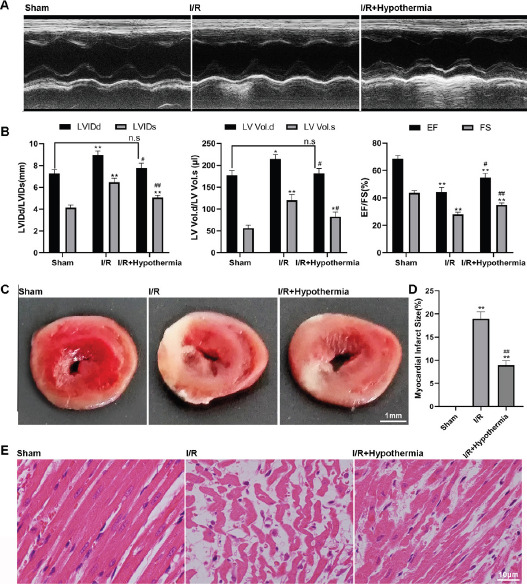
Detection of myocardial function in sham operation group, ischemia reperfusion group and mild hypothermia group. (A) Ventricular space was measured by ultrasonic imaging. (B) Transthoracic echocardiography was used to detect left ventricular related functional indicators: left ventricular end diastolic diameter (LVIDd), left ventricular end systolic diameter (LVIDs), left ventricular end diastolic volume (LV volume. d), left ventricular end systolic volume (LV volume. s), ejection fraction (EF) and left ventricular short ejection rate (FS). (C) TTC staining was used to detect myocardial infarction area. (D) Quantitative analysis of C-chart results. (E) Cardiac collagen fibers were detected by HE staining. n=6, */# p < 0.05, **/## p < 0.01. * It means to compare with the sham operation group, and # means to compare with the ischemia-reperfusion group

Mild hypothermia improves myocardial function and injury in rats with myocardial ischemia-reperfusion. In order to analyze whether mild hypothermia (35°C) can improve the left ventricular function and the degree of myocardial injury in rats after ischemia/reperfusion, we used echocardiography to detect the changes of myocardial function, used 2,3,5-triphenyltetrazolium (TTC) staining to detect the myocardial infarction area, and used hematoxylin eosin (HE) staining to detect the myocardial tissue damage of myocardial fiber degradation. The imaging results showed that the ventricular space of rats with ischemia-reperfusion injury was significantly larger than that of sham operated rats (Figure 1A), but the effect of mild hypothermia significantly contracted the ventricular space. In addition, the obtained left ventricular end diastolic diameter (LVIDd), left ventricular end systolic diameter (LVIDs), left ventricular end diastolic volume (LV volume. d), left ventricular end systolic volume (LV volume. s), ejection fraction (EF) and left ventricular short ejection rate (FS) can more accurately and quantitatively evaluate myocardial ischemia reperfusion and myocardial function damage. Ischemia/reperfusion injury makes the above myocardial function indicators change in a bad direction, while mild hypothermia treatment can make the above indicators develop in a good direction (Figure 1B). The above results indicate that mild hypothermia can significantly improve the myocardial function of rats after ischemia-reperfusion. TTC staining results showed that the myocardial infarction area in sham group was 0, the myocardial infarction area in ischemia-reperfusion group was (18.3±1.9)%, and the myocardial infarction area in mild hypothermia group was (8.7±0.8)%. Tthe myocardial tissue of ischemia- reperfusion injury model rats showed significantly white infarct focus compared with the sham operation group, but the impact of mild hypothermia reduced the details of white infarct focus (Figure 1C, D). The HE staining results showed that the myocardial collagen fibersmyocardial tissue in the ischemia reperfusion injury model rats were significantly damaged compared with those in the sham operation group, and the morphology of myocardial cells was changed, while the myocardial collagen fibersmyocardial tissue in the mild hypothermia group were significantly improved (Figure 1E). TTC and HE staining results showed that mild hypothermia could reduce the risk of myocardial infarction caused by ischemia-reperfusion injury in rats.

Mild hypothermia increases the phosphorylation level of Cx43 and inhibits the transmission of injury signal in myocardial ischemia reperfusion rats

**Figure 2:**
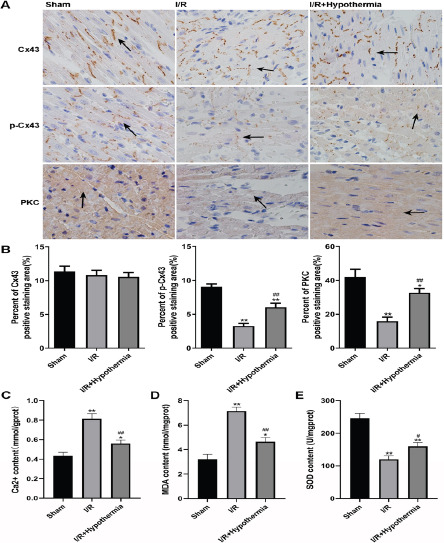
The myocardial injury signal related indexes ot sham operation group, ischemia reperfusion group and mild hypothermia group were detected. (A) The expression of Cx43, p-Cx43 and PKC was detected by immunohistochemistry. The arrows marked in the figure represent the positive part of immunohistochemical staining. (B) Quantitative analysis of results. (C) Detect the content of Ca2+. (D) Detect the content of MDA. (E) Detect SOD activity. n=6, */# p < 0.05, **/## p < 0.01. * It means to compare with the sham operation group, and # means to compare with the ischemia-reperfusion group

In order to determine the effect of mild hypothermia (35°C) on the expression of Cx43 and the signal of injury in rats after ischemia-reperfusion, we used immunohistochemistry and related indicators for analysis. Immunohistochemical results showed that the expression of Cx43 in myocardial tissue of rats undergoing ischemia reperfusion did not change, while the expression of p-Cx43 and PKC in myocardial tissue of rats with ischemia reperfusion injury model was significantly lower than that in sham operation group, but the effect of mild hypothermia made the expression significantly rise, but still lower than that in sham operation group (Figure 2A, B). It shows that mild hypothermia can increase the phosphorylation level of Cx43 in rats induced by ischemia-reper-fusion injury. The detection of Ca2+-content showed that ischemia reperfusion led to the increase of Ca2+content in rat myocardial tissue to 0.81 mmol/gprot, which was significantly higher than 0.43mmol/gprot in the sham operation group, while under the influence of mild hypothermia, Ca2+content decreased significantly to 0.56 mmol/gprot (Figure 2C). The change trend of MDA content is consistent with that of Ca2+, 3.22 nmol/mgprot in the sham operation group, 7.14 nmol/mgprot in the ischemia-reperfusion model group and 4.64 nmol/mgprot in the mild hypothermia group (Figure 2D). The change trend of SOD activity is just the opposite. Ischemia reperfusion led to the decrease of SOD content in rat myocardial tissue to 120.14 U/mgprot, which was significantly lower than 246.38 U/mgprot in the sham operation group. Under the influence of mild hypothermia, the SOD content was significantly increased to 160 U/mgprot (Figure 2E). These results indicate that mild hypothermia can protect cells from injury induced by ischemia-reperfusion.

Mild hypothermia improves cell viability by regulating the phosphorylation level of Cx43 in H9C2 cells with oxygen glucose deprivation recovery

**Figure 3:**
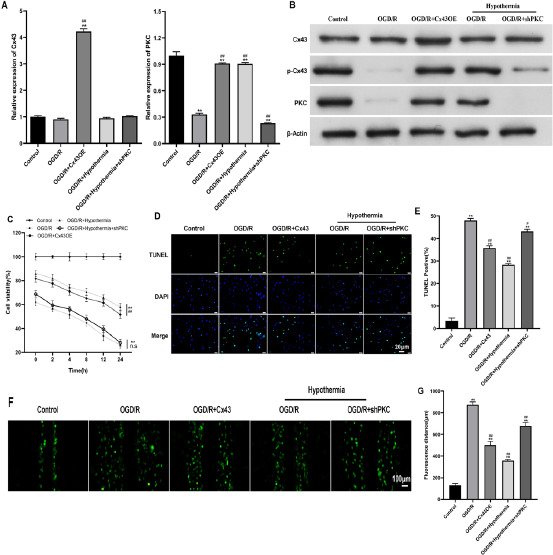
H9C2 cell activity was detected in Control group, OGD/R, OGD/R+Cx43OE group, OGD/R+mild hypothermia group, OGD/R+mild hypothermia+shPKC group. (A) The expression of Cx43 and PKC relative to GAPDH at mRNA level was detected by qPCR. (B) WB detects that Cx43, p-Cx43 and PKC are relative to β- The expression amount of the Actin. (C) The activity of H9C2 cells was detected by MTT method. (D) TUNEL method (scale=20 µm) The content of apoptotic H9C2 cells was observed. The green dot indicates apoptotic cells, and the blue dot indicates DAPI. The stained cells include apoptotic cells and living cells. (E) Quantitative analysis of D figure results. (F) LY method (scale=100 µm) The gap junction permeability of H9C2 cells was observed. The wider the face of the green dot, the higher the permeability of the gap connection. (G) Quantitative analysis of F chart results. */# means P<0.05 and **/## means P<0.01

In order to determine the role of mild hypothermia (35°C) in H9C2 cells, we constructed an oxygen glucose deprivation recovery H9C2 cell model, constructed Cx43OE and shPCK plasmids and transfected them, and detected the expression of Cx43 and PKC in cells under different culture conditions by qPCR and WB. The results showed that at the mRNA level, the expression of Cx43 gene in the group transfected with Cx43OE plasmid was significantly increased, indicating that the construction of Cx43OE plasmid was successful; The expression of PKC gene in the group transfected with shPCK plasmid decreased significantly, indicating that the shPCK plasmid was successfully constructed (Figure 3A). At the protein level, the trend of expression of Cx43 and PKC is consistent with that of mRNA. The expression of p-Cx43 in OGD/R group and OGD/R+shPKC group experiencing mild hypothermia is significantly reduced, and the expression of p-Cx43 in OGD/R+Cx43OE group and OGD/R group experiencing mild hypothermia is significantly restored, close to the control group (Figure 3B), indicating that mild hypothermia can improve the level of p-Cx43 in cells. In order to detect the cell activity, MTT, TUNEL and LY experiments were used for analysis and detection. MTT experiment results showed that the cell viability in OGD/R group and OGD/R+shPKC group experiencing mild hypothermia was significantly lower than that in control group, while the cell viability in OGD/R+Cx43OE group and OGD/R group experiencing mild hypothermia was significantly lower than that in control group, but significantly higher than the above two groups (Figure 3C). TUNEL experiment results showed that the number of apoptotic cells increased after hypoxia, but the number of Cx43OE group and mild hypothermia group decreased significantly (Figure 3D, E). The LY experimental results representing the permeability of the gap connection are consistent with the above TUNEL experimental results (Figure 3F, G). The above reults indicate that mild hypothermia can prevent cell viability decline and apoptosis induced by oxygen and glucose deprivation by increasing the level of p-Cx43.

### Mild hypothermia and Cx43 inhibit apoptosis of H9C2 cells with oxygen glucose deprivation recovery

**Figure 4:**
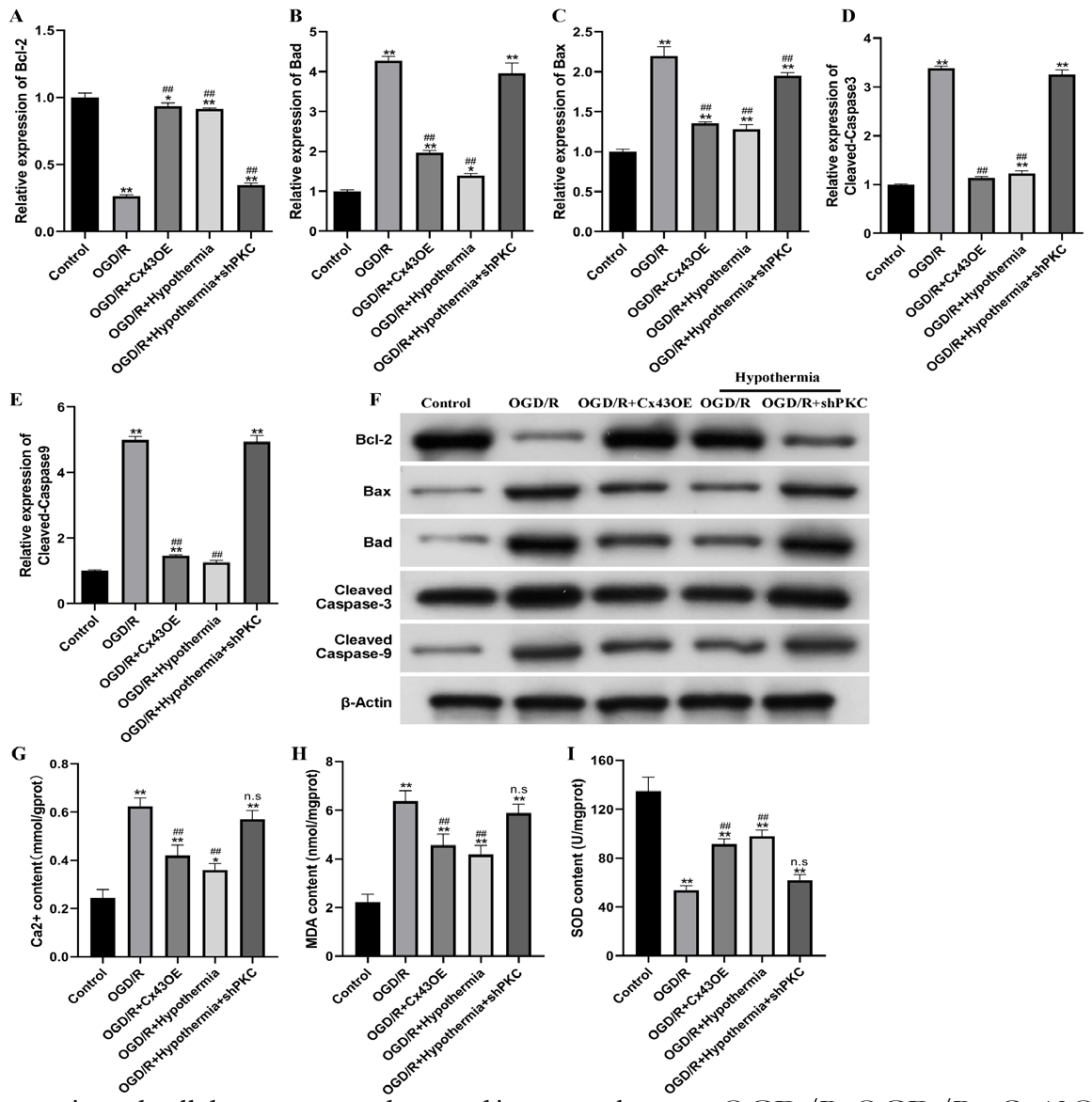
Apoptosis and cell damage were detected in control group, OGD/R, OGD/R+Cx43OE group, OGD/R+mild hypothermia group, OGD/R+mild hypothermia+shPKC group. (A-E) The expression of Bcl-2, Bax, Bad, caspase-3 and caspase-9 relative to GAPDH at mRNA level was detected by qPCR. (F) WB detects that Bcl-2, Bax, Bad, caspase-3 and caspase-9 are relatively β- The expression amount of the Actin. (G) Ca2+content detection. (H) MDA content detection. (I) SOD activity detection. */# means P<0.05 and **/## means P<0.01

The expression of apoptosis related genes at mRNA and protein levels was detected by qPCR and WB. As shown in Figure 4A-F, at the mRNA and protein levels, the expression of Bcl-2 in OGD/R group and OGD/R+shPKC group undergoing mild hypothermia significantly decreased, and the expression of Bax, Bad, caspase-3 and caspase-9 significantly increased, while the trend in OGD/R+Cx43OE group and OGD/R group undergoing mild hypothermia was just opposite. These results indicate that mild hypothermia and overexpression of Cx43 can inhibit apoptosis induced by oxygen glucose deprivation. The detection of Ca2+content showed that OGD/R and OGD/R+shPKC experiencing mild hypothermia led to the increase of Ca2+content in H9C2 cells to 0.62 mmol/gprot and 0.57 mmol/gprot, respectively, which was significantly higher than 0.24 mmol/gprot in the control group, while the Ca2+-content in OGD/R+Cx43OE group and OGD/R group experiencing mild hypothermia decreased to 0.42 mmol/gprot and 0.36 mmol/gprot respectively (Figure 4G). The change trend of MDA content is consistent with that of Ca2+. The control group is 2.23 nmol/mgprot, the OGD/R group is 6.37 nmol/mgprot, the OGD/R+shPKC group experiencing mild hypothermia is 5.88 nmol/mgprot, the OGD/R+Cx43OE group is 4.56 nmol/mgprot, and the OGD/R group experiencing mild hypothermia is 4.18 nmol/mgprot (Figure 4H). The change trend of SOD activity is just the opposite. OGD/R and OGD/R+shPKC experiencing mild hypothermia cause the SOD content in H9C2 cells to decrease to 53.76 U/mgprot and 61.99 U/mgprot respectively, which is significantly lower than the control group 134.9 U/mgprot, while the SOD content in OGD/R+Cx43OE group and OGD/R group experiencing mild hypothermia is increased to 91.37 U/mgprot and 97.94 U/mgprot respectively (Figure 4I). These results indicate that mild hypothermia and overexpression of Cx43 can improve the damage of H9C2 cells caused by hypoxia.

## Discussion

The experimental study of acute myocardial infarction shows that up to half of the final infarct area may be due to reperfusion injury rather than the initial ischemic event^22^,^23^, so the treatment strategy after reperfusion injury is particularly important. In this study, a repeatable, non-invasive and convenient echocardiography was used to detect the cardiac function of SD rats with myocardial ischemia/reperfusion injury. The study showed that after the ischemia/reperfusion injury, the imaging results showed that there was significant myocardial hypertrophy, and various cardiac function indicators of rats were significantly abnormal. Compared with the sham operatin group, LVIDd, LVIDs, LVVd and LVVs measured values were significantly increased, while EF and FS measured values were significantly decreased, EF dropped to less than 45%.

This indicates that the ischemia reperfusion model in this study was successfully constructed. At the same time, under the influence of mild hypothermia (35°C), the ventricular space was significantly contracted, the phenomenon of myocardial hypertrophy was alleviated, and various indexes of cardiac function also tended to develop normally. Compared with the ischemia reperfusion group, LVIDd, LVIDs, LVVd and LVVs measured values were significantly reduced, and EF and FS measured values were significantly increased. The TTC staining of pathological sections also synchronously showed that compared with the sham operation group, the myocardial infarction area after ischemia-reperfusion was significantly increased, while after the introduction of mild hypothermia (35 degrees), the myocardial infarction area was significantly reduced compared with the ischemia-reperfusion group. HE staining showed that compared with sham operation group, myocardial cells in ischemia-reperfusion group were damaged significantly, myocardial collagen fibersmyocardial tissue were dissolved, and inflammatory cells were infiltrated. The introduction of mild hypothermia (35 degrees) could alleviate the damage of myocardial cells. The above results confirm that mild hypothermia can significantly improve the injury caused by myocardial ischemia-reperfusion.

In nature, there are three proteins that can form half channels: connexin, pannexin and innexin. Invertebrates express innexin, and mammals express connexin and pannexin. Among them, pannexin is less expressed in myocardial tissue, while Cx43 in connexin subtype is largely expressed in myocardial cells^24^. Studies have shown that under various stimulation conditions (reduced concentration of divalent cations, alkalization, consistent metabolism, inflammatory factors, dephosphorylation, depolarization, positive membrane potential, hypoxia), the half channel can be opened, and the removal of extracellular calcium ions can increase the diameter of Cx43 half channel from 1.8 nm to 2.5 nm. In myofibroblast, it is proved that ischemia and hypoxia will lead to the closure of gap junctions and the opening of half channels^10^. Mild hypothermia can promote the activity of PKC α It can phosphorylate Cx43 and ε, causing changes in protein conformation, so that gap junctions cannot penetrate fluorescent yellow^25^,^26^. In addition, some studies have found that sufentanil can reduce the myocardial infarction area after ischemia-reperfusion by preserving the level of p-Cx43^27^. Therefore, we detected the expression of PKC and phosphorylated Cx43 under mild hypothermia by immunohistochemistry in vivo and WB experiment in vitro, both of which proved that mild hypothermia can increase the expression of PKC and p-Cx43. The LY experimental research results also verified that mild hypothermia (35°C) can reduce the permeability of gap junctions, causing gap junctions to be unable to penetrate fluorescein.

The results are consistent with the effect of phosphorylated Cx43, blocking the material exchange between cells and the transmission of damage signals.

The mechanism of myocardial ischemic injury is multifaceted. Ischemia leads to the opening of half channels, which leads to myocardial damage, arrhythmia, and intracellular calcium overload. After reperfusion, the injury effect of calcium overload is aggravated. It makes the oxidation function of myocardial cells abnormal, producing a large number of ROS, opening the permeable transition space of mitochondria, activating Caspase, and causing cell apoptosis or necrosis^11^,^28^,^29^. Our research found that after hypoxia, both in vivo and in vitro experiments showed that the content of calcium ions increased significantly, indicating myocardial damage. At the same time, we detected the content of MDA and SOD. The results showed that the oxidation function of cells was abnormal, producing a large number of oxygen free radicals. SOD, as an oxygen free radical scavenger, was largely consumed and destroyed, and the oxidative damage of cells caused by free radicals produced a large number of lipid peroxides. The most important MDA index also confirmed this point after testing.

However, after the introduction of mild hypothermia in vivo experiment, the contents of the three indicators recovered in a good direction, which alleviated the myocardial damage caused by hypoxia reperfusion. In vitro experiment, after the introduction of mild hypothermia and overexpression of Cx43, the content change trend of the three indicators tended to be consistent with that in vivo. When the content of PKC that can phosphorylate Cx43 was artificially interfered, the content change trend of the three indicators tended to be consistent with that of hypoxia. It was speculated that mild hypothermia could alleviate the severe damage to myocardial cells caused by hypoxia by improving the phosphorylation level of Cx43.

In previous studies, it was found that MicroRNA-101 inhibits tgf-β The signal pathway protects myocardial fibroblasts from hypoxia induced apoptosis^30^. TUNEL experiment in this study showed that apoptosis of H9C2 cells was significantly increased after hypoxia and PKC interference, while mild hypothermia and overexpression of Cx43 could reduce apoptosis. In the hypoxia state of adult rat cardiomyocytes and cultured cardiomyocytes, half channel opening leads to myocardial cell damage or apoptosis^31^,^32^, and apoptosis induced by cerebral ischemia also involves half channel dysfunction^33^. Under hypoxic preconditioning, miR133b-5p can protect cardiomyocytes by inhibiting the activation of pro apoptotic proteins such as caspase-8 and caspase-3^34^. Under hypoxia, adrenomedullin inhibits apoptosis of human osteosarcoma cells by up regulating Bcl-2^35^. Therefore, we detected apoptosis related indicators, among which Bcl-2 belongs to anti apoptotic protein, and Bax, Bad, caspase-3 and caspase-9 belong to pro apoptotic protein^36^. Our research results also confirmed this point. At the same time, we found that under the condition of hypoxia and human interference with the content of PKC that can phosphorylate Cx43, the content of apoptosis promoting indicators increased significantly, and the content of anti apoptosis indicators decreased significantly. Under the condition of hypoxia, after the introduction of mild hypothermia and overexpression of Cx43, the above situation can be significantly alleviated, and mild hypothermia can promote the level of p-Cx43, Change the permeability of gap connection. It is speculated that mild hypothermia may regulate apoptosis related proteins by regulating the phosphorylation level of Cx43 to alleviate hypoxia induced cardiomyocyte apoptosis.

## Conclusion

This study aims to clarify that mild hypothermia can participate in the protection of myocardial ischemic injury through Cx43, and provide a new theoretical reference for the treatment of hypoxic injury. However, the specific molecular mechanism of mild hypothermia for the regulation of Cx43 needs further study.

## Conclusion

In this study, we found that mild hypothermia (35°C) can alleviate myocardial ischemia-reperfusion injury in rats by up regulating p-Cx43 level, and reduce hypoxia induced apoptosis of H9C2 cardiomyocytes by changing the permeability of gap junctions. Therefore, mild hypothermia (35°C) may become the treatment of myocardial infarction.

## Figures and Tables

**Table 2:** Comparison of hemodynamic parameters in different treatment groups

hemodynamic parameters	Sham group	I/R group	I/R+Hypotherima group	F	P
LVIDd	7.23±0.46	9.04±0.29	7.65±0.66	46.321	0.012
LVIDs	4.02±0.11	6.37±0.24	5.15±0.10	53.125	0.027
LV volume.d	180.32±17.46	227.42±14.37	173.28±16.51	163.294	0.000
LV volume.s	61.47±8.95	131.56±20.24	65.74±18.26	98.465	0.005
EF	69.21±8.44	44.76±9.71	54.83±8.22	78.748	0.010
FS	43.15±3.26	26.57±4.15	35.67±2.39	63.524	0.013

**Note**: LVID d means left ventricular end diastolic diameter, LVIDs means left ventricular end systolic diameter, LV volume. d means left ventricular end diastolic volume, LV volume. s means left ventricular end systolic volume, EF means ejection fraction and FS means left ventricular short ejection rate
